# Optimization of DNA collection card workflows on the RapidHIT™ ID instrument

**DOI:** 10.1007/s00414-026-03885-z

**Published:** 2026-06-26

**Authors:** Elizabeth J. Kowalczyk, Kapema Bupe Kapema, Jonathan L. King, Robert Lagacé, Michael D. Coble, Jennifer C. Cihlar

**Affiliations:** 1 Center for Human Identification, University of North Texas Health Fort Worth, Camp Bowie Blvd, Fort Worth, TX 3500, 76107 USA; 2 Department of Microbiology, Immunology, and Genetics, University of North Texas Health Fort Worth, 3500 Camp Bowie Blvd, Fort Worth, TX 76107 USA; 3 Human Evolution, Department of Organismal Biology, Uppsala University, Uppsala, 75236 Sweden; 4 Thermo Fisher Scientific, 6065 Sunol Blvd, Pleasanton, CA 94556 USA

**Keywords:** Rapid DNA, RapidHIT™ ID, Ace GlobalFiler™ cartridge, DNA collection cards, STR Profile, Nucleic-card™, EasiCollect™ device, Bode Buccal DNA Collector™

## Abstract

The RapidHIT™ ID System, enables CODIS eligible STR profile generation from reference samples in approximately 90 minutes. While initially optimized for buccal swabs, DNA collection cards, both treated (Nucleic-card™ and EasiCollect™) and untreated (Bode Buccal DNA Collector™), offer alternatives for collection and storage of reference samples. This study evaluated the performance of these cards using ACE GlobalFiler™ Express cartridges across a range of potentially impactful scenarios, including assessment of varying punch sizes, contamination control measures, treated versus untreated collection cards, sample placement within the cartridge, and impact of varying storage times on the collection cards. Results from cross-contamination testing confirmed no detectable contamination between samples, and a 6 mm punch size yielded the highest first-pass success rate and profile quality. Treated cards showed consistent performance during initial runs, regardless of whether they were processed wet or dry. For reruns, the EasiCollect™ cards on average produced higher overall signal when the wetting protocol was used. The untreated Bode card showed greater signal consistency when used dry, while wet treatment improved initial success rates. Proteinase K treatment enhanced DNA recovery from samples aged up to three years. Collectively, these findings provide practical, evidence-based guidelines for implementing DNA collection card-based workflows on the RapidHIT™ ID system, supporting its application across varied forensic and operational environments.

**Supplementary Information:**

The online version contains supplementary material available at https://doi.org/10.1007/s00414-026-03885-z.

## Introduction

The RapidHIT™ ID System produces a DNA profile from biological samples with an approximate 90-minute turn-around time [1]. The system integrates with RapidLink™ and GeneMarker™ software (or GeneMapper™ ID-X for later versions of RapidLink™) enabling short tandem repeat (STR) profile analysis and direct uploads of eligible single-source reference sample profiles to CODIS with NDIS approved Rapid DNA instruments in booking stations or accredited laboratories, as outlined by the FBI [2, 3]. Unlike traditional capillary electrophoresis technologies, this system is user-friendly for operators without scientific backgrounds [2, 4, 5], making it adaptable to non-traditional laboratory settings such as border patrol sectors, state and local booking stations, and active crime scenes [2, 4, 6–8]. Its portability proves useful for (as an example) families of mass disaster victims [9, 10], and can analyze samples including buccal swabs, teeth, blood stains, muscle tissue, and fingernails [11–13]. Further, in laboratory environments, Rapid DNA Analysis can potentially reduce turnaround times for time-sensitive cases, such as ongoing investigations [14, 15].

Currently, RapidHIT™ ID (Applied Biosystems) and ANDE 6 C (ANDE Cooperation) instruments are the only two FBI NDIS-approved rapid DNA instruments for booking station or accredited laboratory reference sample applications [2, 3]. This study focuses on the RapidHIT™ ID instrument using the RapidHIT™ ID ACE GlobalFiler™ Express Cartridge, which is NDIS-approved for processing single-source DNA reference samples, in the form of buccal swabs, in accredited forensic DNA laboratories and booking stations [1–3]. The system provides status indicators to assess profile generation success and identify profiles requiring review [1]. For eligible reference samples, Modified Rapid DNA Analysis must be validated and performed by a qualified analyst who may review flagged samples and determine if they meet the criteria for CODIS upload [2, 3]. Previous research efforts have primarily focused on evaluating and validating the integration of RapidHIT™ ID ACE GlobalFiler™ Express Cartridges using traditional buccal reference swabs [16, 17]. However, DNA collection cards are also widely used in forensic settings/workflows, offering compact and efficient storage for biological samples [18, 19], with ongoing research into usage in Rapid DNA systems [20]. Studies have demonstrated their effectiveness for DNA recovery from various sample types, including bloodstains and buccal cells collected on DNA collection cards [21].

Collection cards come in multiple formats, including treated cards, such as Nucleic-cards™ (Copan) and Whatman EasiCollect™ devices (GE Healthcare Life Sciences), and untreated cards, such as Bode Buccal DNA Collectors™ [22]. Treated cards use chemical coatings to promote cell lysis, protein denaturation, and microbial inhibition, while untreated cards rely on filter paper matrices for DNA preservation [18, 22, 23]. DNA card punchers are used to extract portions of the DNA-containing matrix for testing, with punch sizes varying depending on application requirements and sensitivities [24]. Previous research efforts highlight the value in optimizing workflows for generating profiles from collection card punches on the 3500xL Genetic Analyzer [25, 26]. This study aims to establish optimized guidelines for using DNA collection cards with the RapidHIT™ ID system by evaluating contamination control measures, determining ideal punch sizes, and comparing the performance of treated versus untreated DNA cards. Additional objectives include analyzing the effects of punch wetting, anchoring methods, and sample aging on profile generation. These insights provide practical recommendations for using DNA collection cards with the Rapid DNA instrument.

## Materials and methods

### Sample collection

Samples were collected and anonymized according to North Texas Regional Institutional Review Board (IRB)-approved protocols. Subjects (*n* = 2) were given a study information sheet with details specific to the study. A waiver of the requirement to obtain written consent was requested and obtained from the North Texas Regional IRB. All four sub-studies (described in more detail below) used the same two subjects, one male and one female, each of which donated multiple samples. The number of samples collected from each subject was approximately equal, with minor variation resulting from the need to rerun certain samples (reruns described in more detail below). Nucleic-cards™, Whatman EasiCollect™ Devices, and Bode Buccal DNA Collectors™ were used in this study following the manufacturer recommended collection protocols [22, 27–29] for each type of DNA collection card. For this study’s purpose, a swipe was deemed one motion from the top of the cheek to the bottom. Each card collection was spaced at least four hours apart, collected 30 minutes after rinsing the mouth and refraining from eating or drinking. After collection, cards were allowed to dry for at least 24 hours before punching.

### RapidHIT™ ID processing

A total of 205 runs (including positive and negative controls) were processed on the RapidHIT™ ID instrument in a series of studies (described below) designed to optimize DNA collection card processing on the RapidHIT™ ID instrument (RapidHIT™ Software v2.0) with the ACE GlobalFiler™ Express Sample Cartridge and RapidLINK™ software (v2.0) workflow. DNA collection cards come in different shapes; therefore, sampling approaches can differ. Treated cards (EasiCollect™ Devices and Nucleic-cards™) have a circular sample collection area and were sampled using a Harris Uni-Core Puncher (Ted Pella, Inc.) to generate circular cut-outs referred to as “punches”. Untreated cards (Bode Buccal DNA Collector™) have an elongated shape and were cut into 5 mm wide sections (per the manufacturer recommendation), referred to as “strips” throughout this study. All manufacturer recommendations were followed when operating the RapidHIT™ ID instrument, referred herein as “Standard GFE ACE protocol.” At the end of each run, the cartridge was removed, and the DNA punch or strip was dried and stored at room temperature for potential re-analysis. To test whether DNA punches could be analyzed more than once, we physically removed a punch from its original used cartridge, placed it into a new unused cartridge, and processed it again on the RapidHIT™ ID instrument. The first analysis of a fresh punch in a new cartridge is the “initial run,” and the subsequent analysis of the same punch in a new cartridge is referred to as the “rerun”. The goal was to evaluate whether a single punch could still produce a usable DNA profile when transferred into a fresh cartridge and reprocessed.

### Cross-contamination detection and punch size optimization

To assess potential cross-contamination and identify the optimal punch size, Nucleic-cards™ were sampled at three punch sizes: 3 mm, two 3 mm, and 6 mm (Supplemental Fig. 1). A total of 38 runs were completed. Punches were collected and run with the following sequence (for each punch size): Female, Male, Blank, Male, Female, Blank, Female, Male, Blank, Male, Female, Blank). A Harris Uni-Core Puncher (Ted Pella, Inc.) was used to collect a punch from an unused card after each of the samples listed above to effectively “clean” the punch between samples. A 10% bleach solution was used to wipe the pad before each sampling. Each collected punch was prepared by adding 30 µL of distilled water, followed by spearing with a lancet and immediate transfer to the GFE ACE cartridge. The punches were placed at the bottom of the swab loading tube, and samples were processed using the Standard GFE ACE protocol.

### Performance evaluation of treated DNA cards

Punches from Nucleic-cards™ and EasiCollect™ devices were collected and prepared using two conditions: wet punches (treated with 30 µL of distilled water) and dry punches. Based on previous results from the punch size optimization study, only the 6 mm punch size was used. Wetted samples were anchored by a lancet and transferred to a cartridge. Dry punches were either anchored by a lancet or placed with no anchor into the cartridge (Supplemental Fig. 2; a swab was used to push the punch to the bottom of the cartridge and then removed). As described above, at the end of the initial run, a subset of punches was removed from their used cartridge, placed into a new cartridge, and processed again on the RapidHIT™ ID system, with these repeat analyses referred to as “reruns” (the same punch analyzed twice). In this re-evaluation, sixteen punches from each card type were taken and run under the two best performing conditions (n = 8 for each condition). This resulted in 32 reruns, which, together with the initial runs and controls, brought the total to 83 runs in this sub-study.

### Performance evaluation of bode buccal DNA collector™ (Untreated DNA Card)

Filter paper from the collectors was cut into three 5 mm strips corresponding to the left, middle, and right sections of the collection device, with edge pieces being defined as the left and right sections, (use of the terms “left”, “middle”, and “right” throughout the manuscript refers to which portion of the filter paper was used for analysis and not where the paper was placed in the sample cartridge) (Supplemental Fig. 3 (A)), with strips divided between dry and wetting protocols. Each treatment was further subdivided into three anchoring methods: (1) “with swab” - secured with a swab that remained in place for the entity of the run (Supplemental Fig. 3 (B)); (2) “no swab” - pushed to the cartridge bottom with a swab that was then removed, where the strip remained in the cartridge without the swab present during the run; (3) “with lancet” - speared with a lancet that remained in place for the entire run. Samples were processed on the RapidHIT™ ID system using the standard GFE ACE protocol. If a first-pass run failed (e.g., produced a red “X” or generated a major error such as “SRI2”), the same strip was removed and processed under the same treatment, anchoring method, and location, as described above. These repeat attempts were to assess whether a failed sample could give an improved or complete profile when run a second time. In addition, six samples were “rerun”, two for each location (left, middle, right) under the “no swab dry” condition, to evaluate whether rerunning the same strip could continue to produce a complete profile. In this sub-study, there were 48 total runs, which included the initial runs, reruns, and controls.

### Aged samples

Samples were aged on three different card types for three different time intervals: fresh, intermediately aged, and long-term storage aged. Fresh samples were run within a few days of collection, with these runs completed as part of the studies described above. Intermediately aged samples were stored for an average of 1.5 years, ranging from 1 year 4 months to 1 year 9 months post sample collection. Long-term storage aged samples were stored for an average of 3 years, ranging from 2 years 11 months to 3 years 3 months post sample collection. To age the samples, they were collected as described in Sample collection and stored at room temperature (laboratory is set to approximately 22 degrees Celsius) in standard manila envelopes. The envelopes were placed in a drawer within the laboratory. Relative humidity was not actively controlled, and the envelopes were not airtight in order to allow passive airflow and reduce moisture accumulation, reflecting case scenarios where no additional measures were taken to preserve the sample as the flexibility of Rapid instruments may involve workflows where minimal equipment and storage options are available. All three intervals were run with no treatment (i.e., dry and no anchoring method) to establish a baseline of comparison for the treatments. Intermediately aged Nucleic-cards™ had 4 treatment groups: 10 µL proteinase K (Prok), 50 µL Prep and Go Buffer (Png) incubated at 56 °C for 60 min, 50 µL Png plus 10 µL Prok incubated at 56 °C for 60 min, and double punched (two punches instead of one) with no additional treatment. Proteinase K treatments used commercial Proteinase K at a concentration of ~ 20 mg/mL (pulled from the QIAamp DNA Investigator Kit for the study herein). Following any post-treatment and incubation, punches were not air-dried and were transferred directly into the ACE sample cartridge for processing. Based on the results of the Nucleic-cards™, only the 10 µL Prok treatment and the 50 µL Png plus 10 µL Prok incubated treatments were used for the other intermediately aged card types. The best treatment for each card type was determined (through evaluation of performance metrics described below) and then applied to long-term storage samples, with a total of 36 runs.

### Concordance data from 3500 genetic analyzer workflow

DNA was extracted from buccal swabs using the PrepFiler™ Forensic DNA extraction kit [30] as per manufacturer’s guidelines. The extracted DNA was quantified by qPCR using the Quantifiler™ Trio DNA Quantification kit [25, 31] on a 7500 real-time PCR system [32] following manufacturer’s guidelines. Extracted DNAs were amplified with the GlobalFiler™ Express PCR Amplification Kit [32] on a Veriti^®^ Thermal Cycler (Thermo Fisher Scientific) as described in Cihlar et al. 2022 [17]. The amplified products were subjected to electrophoresis on a 3500xL Genetic Analyzer (Thermo Fisher Scientific) according to the GlobalFiler™ Express PCR Amplification Kit User Guide [32]. Raw data were analyzed using GeneMapper™ ID-X Software v1.6 (Thermo Fisher Scientific), with allele peak threshold calls set at 175 relative fluorescence units (RFU).

### Data analysis

All of the 205 runs were processed on the RapidHIT™ ID instrument using RapidHIT™ ID Software v2.0, Expert System v2.0, the ACE GlobalFiler™ Express Sample Cartridge, and the RapidLINK™ Software v2.0 workflow. Throughout the study, RapidLINK™ Software was used for tracking runs, instrument performance, and consumables [33]. The RapidHIT™ ID system uses a single-capillary electrophoresis setup; therefore, it cannot co-run an allelic ladder with the sample. To address this, a library of allelic ladders (each generated under distinct migration conditions) is employed for allele sizing and identification [34]. For each sub-study, performance metrics, including first-pass success rate, peak heights, and peak height ratios (PHR), were collected. The first/primary analysis was performed using GeneMarker^®^ HID STR Human Identity Software (HIDAutoLite v2.9.5; SoftGenetics, State College, PA, USA), integrated within the RapidLINK™ Software. Data extracted from the peak table output file included allele calls, PHR, peak heights, and the presence and types of quality flags [35]. Analytical and stochastic thresholds used in this study were pre-set in the software according to Thermo Fisher Scientific’s validation data [1]. For the ACE GlobalFiler™ Express Sample Cartridge, the analytical threshold was set to 35 RFU [1]. For stochastic threshold, the manufacturer specifies loci specific stochastic thresholds ranging from 35 RFU to 140 RFU [1]. Since a range of stochastic thresholds are used, 140 RFUs was chosen to conservatively represent the stochastic threshold in the relevant figures included in this study. For each locus, “Total Signal” was calculated as the sum of the allele peak heights (RFU) observed at the locus. Peak heights were notated by the software and collated regardless of whether they fell below the analytical threshold of 35 RFU, to provide an overall assessment of profile signal across all loci. For each run, the average total signal was calculated as the mean of the locus-level Total Signal values across all loci in that profile. As locus-level values were averaged across a run (average Total Signal per run), they were not treated as independent observations for any subsequent statistical calculations. A first-pass success was defined as the generation of a complete STR profile with no software quality flags, indicated by a green check mark at run completion. Samples with quality flags (indicated by a yellow check mark) underwent a secondary manual review (termed “Modified Rapid DNA Analysis”) by a trained scientist. A profile was not modified if any alleles were missing, even if the allele appeared present upon visual inspection, as the peak fell below the established thresholds. In this particular study, the only circumstances in which a profile was modified (quality flag or allele call removed) was when a profile appeared to be complete and single-sourced with only a single heterozygous locus displaying a peak imbalance quality flag or when a known dye artifact was identified. All statistical analyses were evaluated using a significance level of α = 0.05. Statistical tests are reported in line with each comparison and included one-way ANOVA for multi-group comparisons and two-sample t-tests for independent group comparisons. Reported p-values reflect per-run comparisons with an assumption of normality, where very small p-values are expressed using threshold notation (e.g., *p* < 0.0001). Statistical analyses and data handling were conducted using Microsoft Excel and R (v4.4.1) with RStudio. BioRender was used in part to help represent the modified changes in Fig. 3.

## Results and discussion

Results are presented from a series of studies designed to establish optimized guidelines for using DNA collection cards with the RapidHIT™ ID system, providing guidance for the wide array of potential operators of Rapid DNA instruments. This included assessments on cross contamination detection, optimal punch size, overall performance for treated and untreated cards, and samples aged from intermediate to long term storage length.

### Cross-contamination detection and punch size optimization

This sub-study investigated the potential for sample-to-sample carryover when processing DNA collection cards with the RapidHIT™ ID System using the ACE cartridge, assessing the impact of processing an additional blank punch for contamination control with the Nucleic-card™. This study also assessed the optimal punch size for DNA collection cards for downstream analysis.

STR profiles were successfully generated for all samples in this sub-study. Worth noting during this sub-study, both blank and DNA-positive samples displayed a known artifact [36] at the AMEL locus (below the analytical threshold), with only two blank punch cartridges registering an allele call. These allele calls were flagged as Out-of-Bin (OB) peaks at the AMEL locus [31, 36]. Supplemental Fig. 4 illustrates examples of a run blank with an uncalled artifact, a run blank with an OB-labeled artifact, and a DNA-positive sample with an uncalled artifact. This artifact was noted during manual review of the STR profiles but did not impact downstream analyses.

Concordance data for the two study subjects indicated 38 total alleles for the female participant and 42 alleles for the male participant across all loci tested. The number of potential alleles was calculated by adding the number of shared alleles (17) with the alleles unique to each contributor (21 female; 25 male), resulting in 63 potential alleles. Supplemental Fig. 5A provides an example of this allele mixture. Cross-contamination (or sample-to-sample carryover) was evaluated by examining STR profiles and blanks for evidence of allele carryover. No alleles unique to the male profile were detected in female samples, no alleles unique to the female profile were detected in male samples, and no alleles were called for the blank punches, demonstrating the absence of cross-contamination and the effectiveness of the cleaning protocol used. Supplemental Figures (5B and 5C) show examples of full, uncontaminated STR profiles for both participants.

**Fig. 1:**
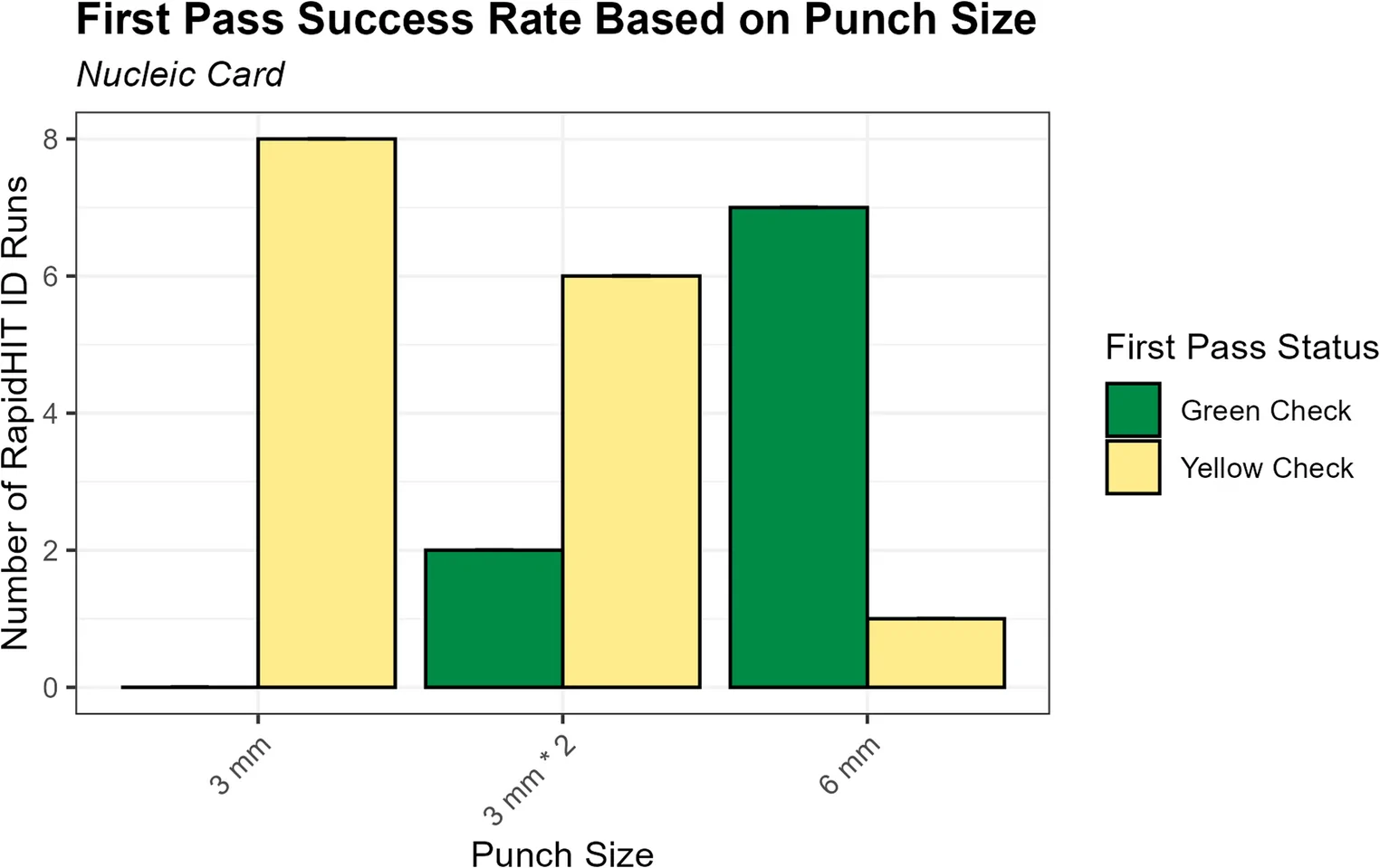
First-pass success rates (indicated by color) for each punch size (x-axis) are shown. Green bars represent runs without any quality flags, while yellow bars represent runs with at least one flag. The data are segmented by punch size diameters of: 3 mm, 3 mm × 2, and 6 mm, corresponding to surface areas of, 28.26, 56.52, and 113.04 sq. mm respectively

The effect of punch size on generating a complete profile was analyzed using eight runs per punch size. As shown in Fig. 1, larger punch sizes corresponded to improved first-pass success rates, characterized by fewer flagged profiles (yellow checks) and more unflagged profiles (green checks). Results showed 0% of the 3 mm samples, 25% of the 3 mm × 2 samples, and 87.5% of the 6 mm samples generated complete, quality flag free profiles.

**Fig. 2:**
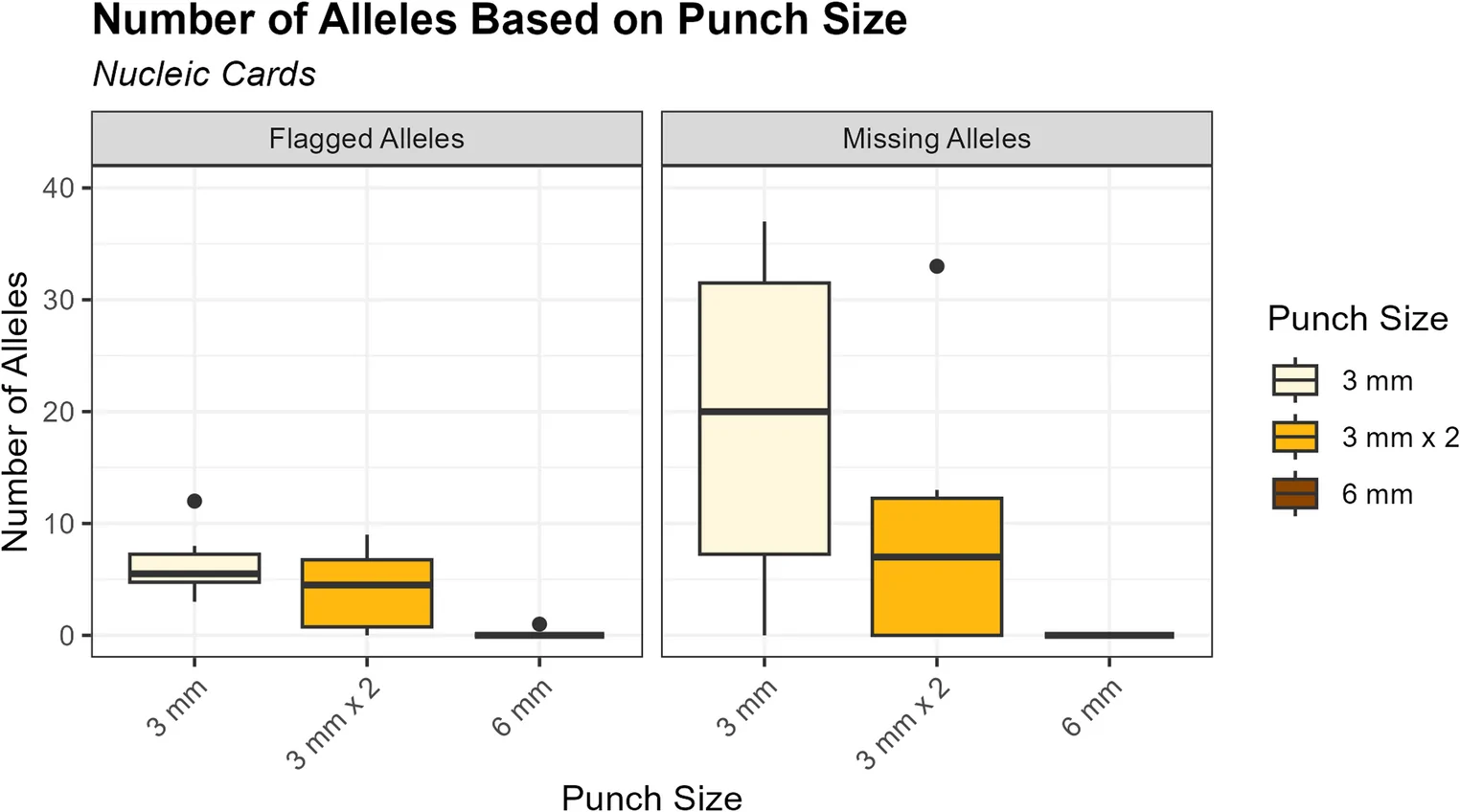
Box plots illustrating the distribution of flagged alleles and missing alleles (y-axis; faceted by category) by punch size (x-axis and color; 3 mm, 3 mm × 2, and 6 mm) as identified by the RapidHIT™ ID system

Figure 2 shows a decrease in the number of flagged and missing alleles per run as punch mass increased. The 6 mm punch size consistently produced the best results, with only one flagged allele in a single sample. In contrast, the 3 mm punch had a mean of 5.4 flagged alleles (SD = 1.7) with an outlier of 12, while the 3 mm × 2 punches averaged 4.3 (SD = 3.7). Missing allele counts followed the same pattern where the 6 mm punch had no missing alleles, while the 3 mm punch averaged 19.3 (SD = 13.4) and the 3 mm × 2 punches averaged 5.6 (SD = 5.9) (with a single outlier (value = 33)) missing alleles. These differences were statistically significant when comparing per-run counts across punch sizes, with both flagged alleles (ANOVA, *p* = 0.002, *n* = 8) and missing alleles (ANOVA, *p* = 0.003, *n* = 8) varying across punch sizes. Based on these results, the 6 mm punch size was selected for subsequent studies.

### Performance evaluation of treated DNA cards

**Fig. 3:**
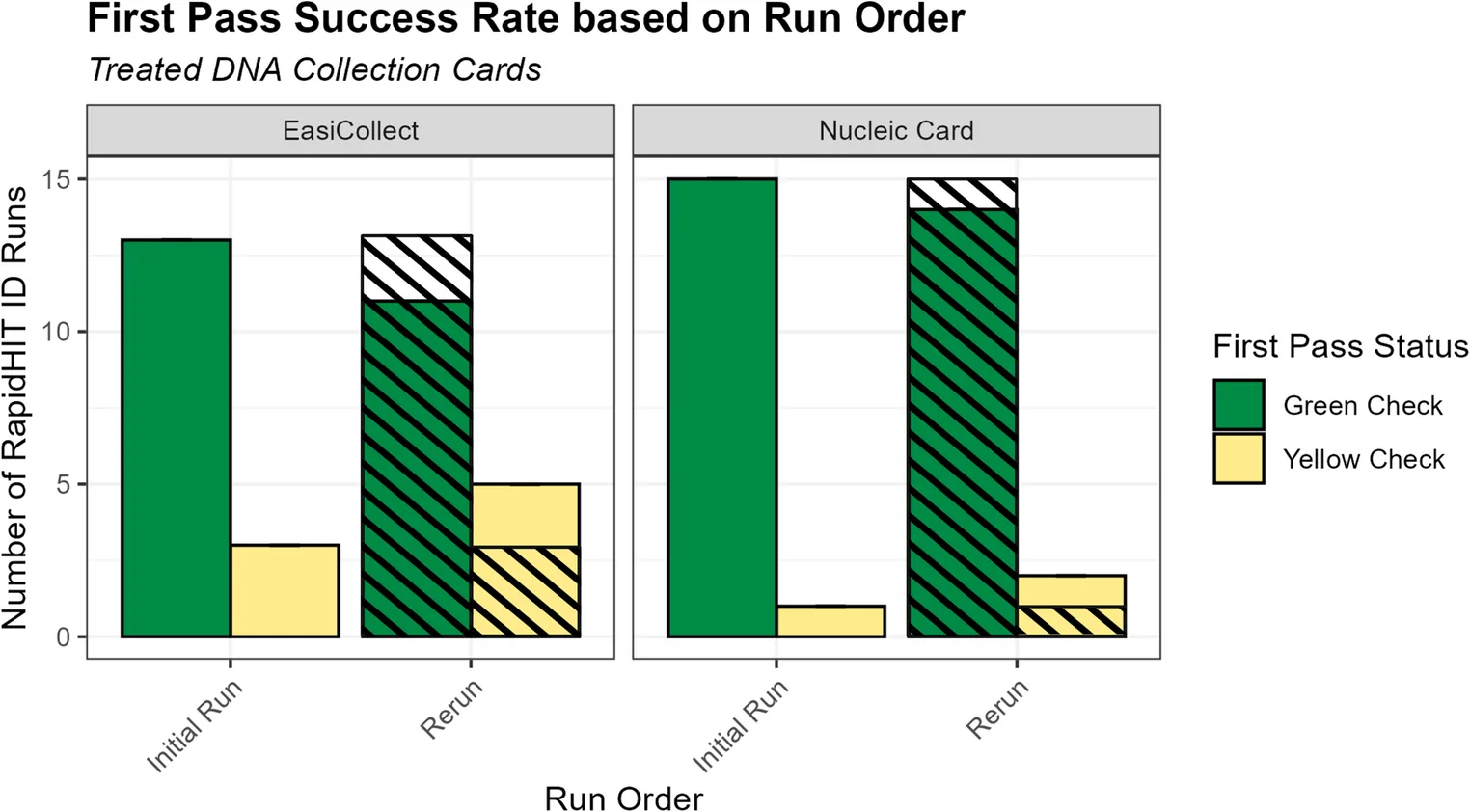
Bar graphs illustrating the first pass success rates (indicated by color) for the initial run and rerun (x-axis) for EasiCollect™ Devices and Nucleic-cards™ (facets/columns). Green bars indicate runs with no quality flags, while yellow bars indicate runs with at least one flag. Diagonal lines, overlaid in BioRender, represent the modified first pass success rate after manual review

This sub-study assessed the performance of treated DNA collection cards (Nucleic-card™ and EasiCollect™) with the RapidHIT™ ID system, focusing on treatment (wetting versus no wetting), anchoring (lancet versus no lancet), and rerun capabilities. Figure 3 shows the first-pass success rates for the EasiCollect™ card and Nucleic-card™, with wet-with-lancet and dry-with-lancet results pooled. The data are further separated by initial run and rerun (secondary run of the same punch in a new ACE GlobalFiler™ Express Sample Cartridge), with Modified Rapid Analysis results indicated by horizontal lines. During the initial run, the Nucleic-card™ (Fig. 3) had 15 passes and 1 flagged run. Reruns had 14 passes and 2 flagged runs, with one yellow-flagged profile reclassified as green after Modified Rapid Analysis. The EasiCollect™ card (Fig. 3) on its initial run had 13 passes and 3 flagged runs initially, and 11 passes and 5 flagged runs on rerun. Two of the rerun yellow profiles were reclassified as green after Modified Rapid Analysis. Lower first-pass success rates in reruns were expected, as the amount of DNA on the punch is partially consumed during the initial run. In all three reclassifications, the profiles had a single flag, indicating a peak imbalance at one locus in otherwise single-source samples. Further, Supplemental Fig. 6 shows the number of missing and flagged alleles across initial runs, non-modified reruns, and modified reruns. Modified Rapid Analysis resulted in comparable first pass success rates for both the initial and secondary runs, highlighting the value of manual review by a qualified analyst (Fig. 3).

**Fig. 4:**
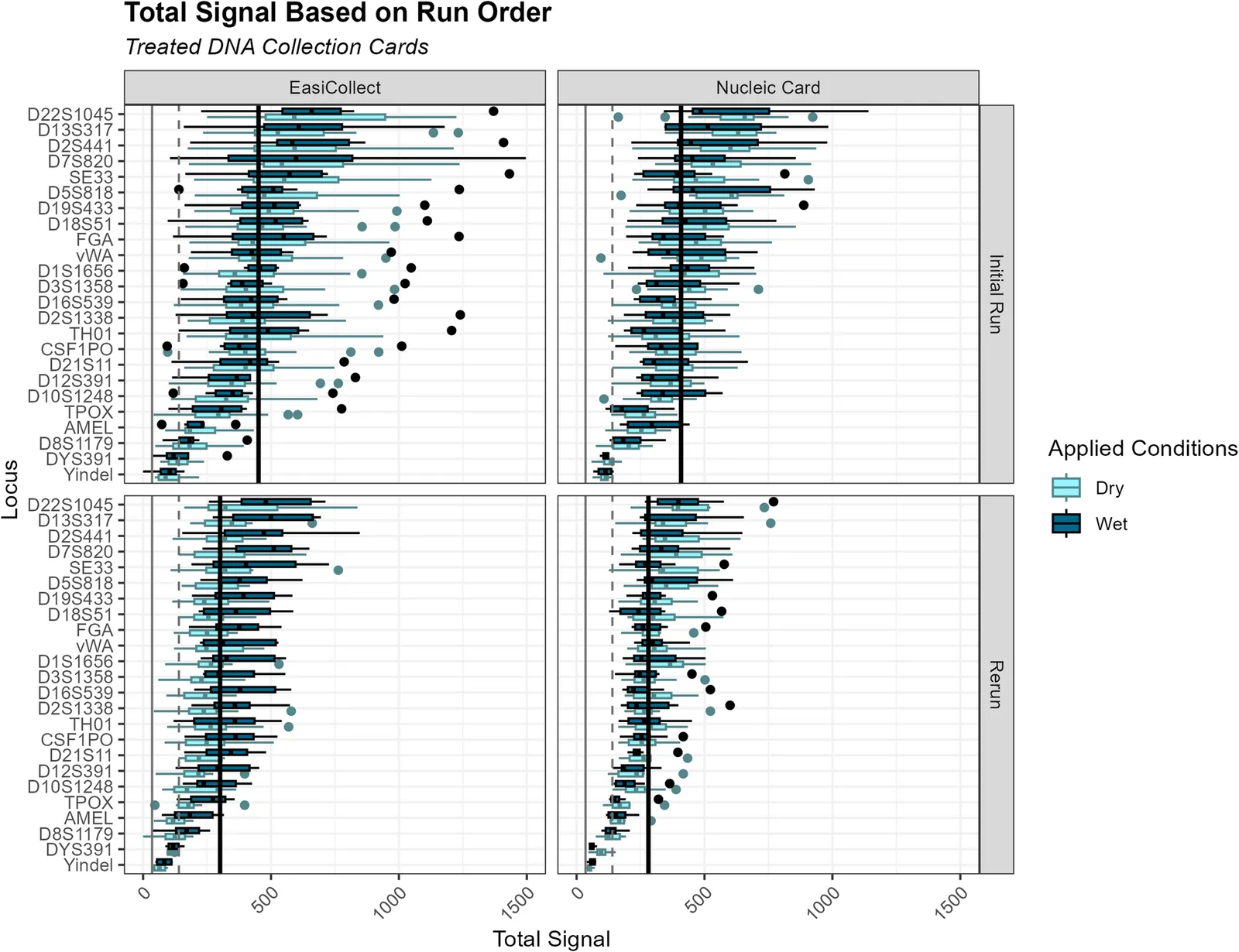
Box plot of total signal (x-axis) at each locus (y-axis) for both treated card types (facets; columns) by treatment (color) and run order (facets; rows). The vertical gray bars show the analytical at 35 RFU (solid gray line) and stochastic thresholds at 140 RFU (dotted gray line). The vertical solid black bar represents the average total signal per run

Figure 4 highlights that initial runs yielded higher average total signals per run than the reruns. This trend is consistent with previous studies [17] and not unexpected as some of the DNA on the punch is consumed with each subsequent run on the RapidHIT™ ID instrument. Both cards consistently produced usable DNA profiles above analytical (35 RFU) and stochastic (91–140 RFU) thresholds. The average total signal per run for the EasiCollect™ card was 451.3 (SD = 127.0) for the initial run and 301.0 (SD = 75.2) for the rerun, and for the Nucleic-cards™, average total signal per run was 408.5 for the initial run (SD = 109.9) and 280.8 (SD = 71.7) for the rerun. Wetting appeared to mitigate signal loss for EasiCollect™ reruns, yielding average total signal per run of 348.8 (SD = 76.2) for wet versus 253.2 (SD = 70.4) for dry (t-test comparing average total signal per run, *p* < 0.0001, *n* = 8), whereas no significant effect of wetting was observed for the Nucleic-card™ reruns (t-test comparing average total signal per run, *p* = 0.17, *n* = 8).

Supplemental Figs. 7 show initial first-pass success rates under varying conditions. The Nucleic-card™ achieved 100% first pass success when dry and anchored with a lancet, with one flagged run each for the dry/no-lancet and wet/lancet conditions (Supplemental Fig. 7). The EasiCollect™ card achieved 100% first pass success when dry and not anchored, with two flagged runs for the dry/lancet condition and one flagged run for the wet/lancet condition (Supplemental Fig. 7). Examining the reasons for quality flags, Supplemental Fig. 8 shows the number of missing and flagged alleles for each card. Most quality flags were due to a single missing or flagged allele, with the highest number being two flagged alleles; otherwise, profiles were complete. Further, Supplemental Fig. 9 (EasiCollect™ Devices) and Supplemental Fig. 10 (Nucleic-card™) show no clear pattern in average total signal per run by anchoring or treatment.

Overall, both cards performed comparably during initial runs, with the respective reruns consistently producing profiles above the thresholds. This means that these punches from collection cards loaded with valuable samples, can be saved and run at a later time, run on a different workflow, provided to another laboratory for analysis, etc. While a small number of the rerun profiles (*n* = 2 for Nucleic-card™; *n* = 5 for EasiCollect™ card) had quality flags, some of these could be resolved by a modified rapid analysis, highlighting the value of manual review by a qualified analyst. Wetting improved the average total signal per run for EasiCollect™ reruns but added complexity to the workflow with no impact on initial runs. The lancet improved punch placement, ensuring the punches remained submerged in the lysis buffer, and ease of use (i.e., picking up the small punch and placing it in the cartridge). However, the lancet provided no appreciable impact to total signal and first pass success rate.

### Performance evaluation of bode buccal DNA collector™ (Untreated DNA Card)

This study evaluated the performance of the Bode Buccal DNA Collector™ using the RapidHIT™ ID system, focusing on three variables: treatment (wetting versus no wetting), anchoring method (lancet versus swab versus no swab), and sample location (left versus middle versus right). One failed run occurred with a left-section strip held down with a lancet under wet treatment. The punch was removed and rerun in a new cartridge under the same conditions (wet treatment with lancet) and produced a green profile. The cause of the initial failure was likely a blocked channel as it received an “SRK2” error code and still had lysate present in the sample cartridge when removed from the instrument (Supplemental Fig. 11).

Further, five samples triggered an SRI2 error, one of which failed on both the initial run and the rerun, resulting in six total runs with the error. An SRI2 error indicates an issue arose during sizing that could affect accurate allele calling [17]. Of the 5 initial runs, two were rerun (same strip was removed from the ACE sample cartridge and placed in a new ACE sample cartridge), where one sample improved from yellow to green and one remained yellow with the SRI2 error. When all six runs that received the SRI2 error were reprocessed using the current instrument software V10.4.0.10, while the original data was processed using V10.1, five produced complete and accurate profiles. The addition of a new systemic ladder library enabled the samples to be correctly sized for genotyping. In the remaining case, the SRI2 error was resolved, but other quality flags kept the profile at yellow.

Table 1 shows the first pass success rates after the single red profile was removed for treatment, sample location, and anchoring method. In the first pass runs (not shown in table), wet treatment produced 84.2% green, 10.5% yellow, and 5.2% red profiles, while dry treatment yielded 72.2% green, 27.8% yellow, and 0% red. When the single red-profile wet sample was rerun and produced a green check profile, the wet treatment’s adjusted pass rate increased to 89.5% green and 10.5% yellow, with no reds. While this suggests a first pass rate advantage for wet treatment, the cause of the red profile leaves open the possibility that wetting combined with certain anchoring methods could contribute to failures.

**Table 1:** Direct comparison of first-pass success rates following a single rerun, as discussed in the text, across treatment conditions (wet or dry), punch location (right, left, edge, or middle), and anchoring method (swab, lancet, or no swab). Values are presented as percentages of profiles with a pass status and profiles with quality flags. Categories associated with rerun conditions are further described in the text*

		Pass	Quality flag
Treatment	Wet *	89.5%	10.5%
	Dry	72.2%	27.8
Location	Right	69.2%	30.8%
	Left *	91.7%	8.3%
	Edge *	80.0%	20.0%
	Middle	83.3%	16.7%
Anchoring	With Swab	76.9%	23.1%
	Lancet *	91.7%	8.3%
	No Swab	75.0%	25.0%

For sample location, the right section had 69.2% green and 30.8% yellow, with no reds. The left section had 83.3% green, 8.3% yellow, and 8.3% red. Grouping the left and right as “edge” samples yielded 76% green, 20% yellow, and 4% red. The middle section produced 83.3% green and 16.7% yellow, with no reds. When the left-section red profile was removed and rerun, producing a green check (mentioned above), the left section’s adjusted pass rate became 91.7% green and 8.3% yellow, and the edge grouping rose to 80% green and 20% yellow, making it more comparable to the middle section’s 83.3% green and 16.7% yellow.

For anchoring methods, first-pass results showed: “with swab” having 76.9% green, 23.1% yellow, 0% red; “with lancet” having 83.3% green, 8.3% yellow, 8.3% red; and “no swab” having 75% green, 25% yellow, 0% red. When the red-profile lancet sample was rerun, producing a green check (mentioned above), the adjusted lancet pass rate became 91.7% green and 8.3% yellow. While this improved the lancet group’s first pass success rate, it remains possible that lancet anchoring of a wet strip could block the channel opening, which could explain the initial red failure.

In total, six strips/punches (two from each section of the Bode Collector) were selected for rerun testing under the same conditions to assess the potential ability of running the same strip more than once. Four were green on the initial and secondary run, while two produced yellow checks on the initial run with one green and one yellow check on its secondary run. As with the treated collection cards, this demonstrates that the Bode Collector strips can be saved and rerun if necessary or desired.

**Fig. 5:**
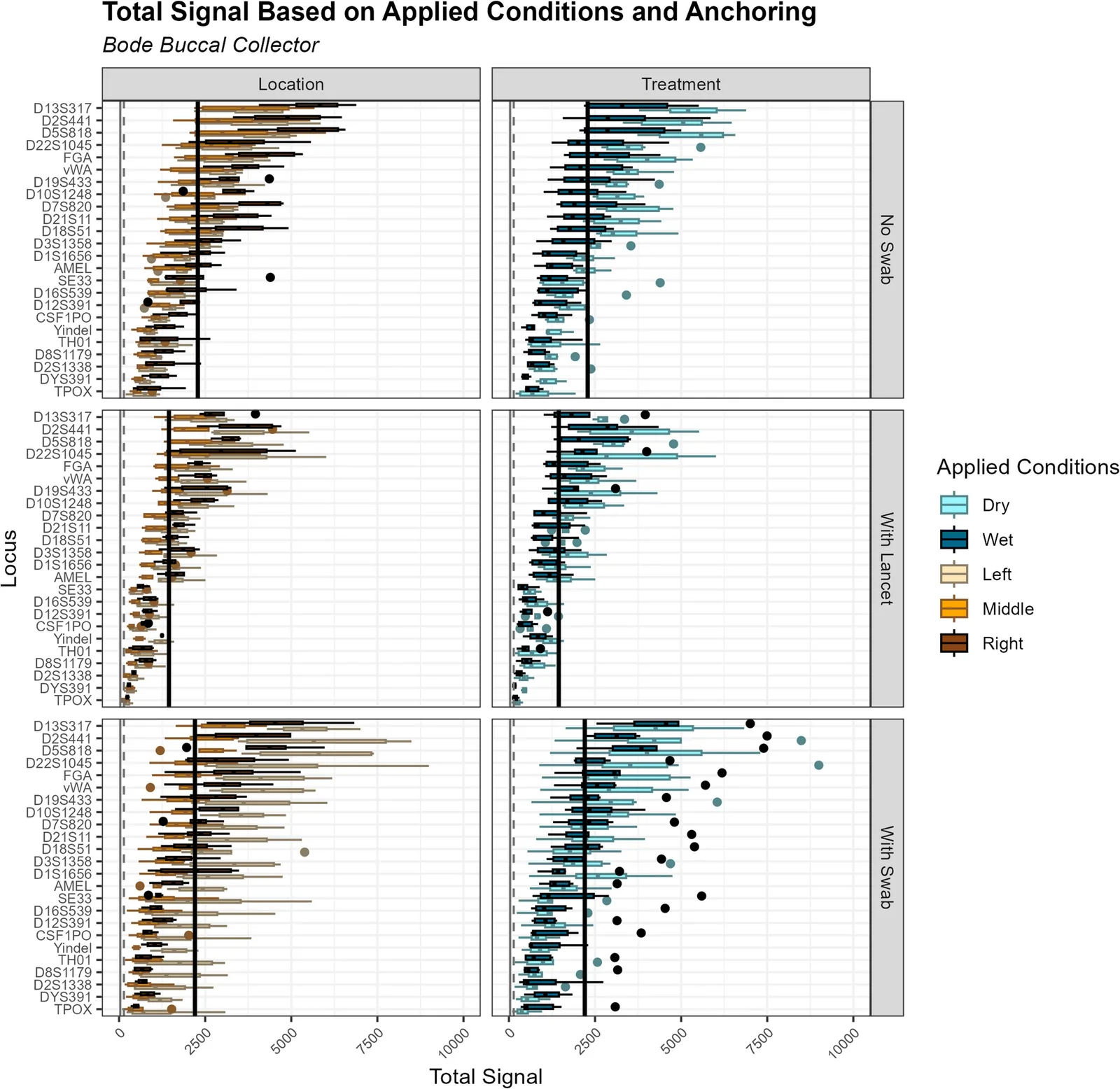
Total signal (x-axis) based on applied conditions (facets; columns) and anchoring (facets; rows) box plots. Loci are indicated on the y-axis. The runs that received the wetting protocol are dark blue, and the dry samples are light blue. Samples taken from the left section are pale yellow; the middle section samples are orange, while the right-side samples are dark brown. The vertical gray bars show the analytical at 35 RFU (solid gray line) and stochastic thresholds at 140 RFU (dotted gray line). The vertical solid black bar represents the average total signal per run

Figure 5 compares the effects of treatment, sample location, and anchoring method on total signal. Table 2 directly compares average total signal per run between the location and treatment, while Table 3 directly compares the anchoring method to the treatment. Dry treatment produced higher or equivalent average total signal per run across all locations. The middle and right sections of the Bode Collector with the dry treatment had higher averages than the wet treatment counterparts (Table 2). The left section had nearly identical average total signal per run in regard to its treatment. The middle section had the lowest average total signal per run, regardless of the treatment, a trend which was also seen in Carter et al., where the edge pieces had better quality profiles than the middle section [37]. Similarly, dry treatment produces higher or equivalent total signals across all anchoring methods. The “with lancet” condition had the lowest average total signal per run for both wet and dry treatments. The “with swab” condition exhibited the least differences between wet and dry treatments, with total signal averages of 2244.65 and 2273.08 respectively.

**Table 2:** Direct comparison of average total signal per run between location (left, middle, right) and treatment (wet versus dry). Values are presented as average total signal per run and standard deviation (SD) for each condition

	Wet treatment		Dry treatment		Total	
	Average	SD	Average	SD	Average	SD
**Left**	2428.46	988.39	2431.59	1266.75	2430.17	1115.05
**Middle**	1262.80	581.47	1733.63	934.11	1498.21	735.54
**Right**	1884.30	987.74	2605.45	1324.76	2244.88	1127.12
**Total**	1825.00	826.37	2256.89	1143.71

**Table 3:** Direct comparison of average total signal per run between anchoring method (with swab, no swab, with lancet) and treatment (wet versus dry). Values are presented as average total signal per run and standard deviation (SD) for each condition

	Wet treatment		Dry treatment		Total	
	Average	SD	Average	SD	Average	SD
**With Swab**	2244.65	985.59	2273.08	1252.71	2258.86	1066.10
**No Swab**	1889.45	848.77	2810.28	1332.36	2349.86	1063.26
**With Lancet**	1244.06	741.82	1687.33	993.49	1466.44	858.41

Overall, dry treatment produced higher average total signals per run compared to wet treatment (t-test comparing average total signal per run, *p* < 0.0001, *n* = 18). Dry treatment had a comparable first-pass success rate, with most yellow checks attributable to four runs affected by the SRI2 error (*n* = 18; 4 with SRI2 error, 1 with a single missing and flagged allele). Wet treatment achieved similar first-pass success rates but lower total signals and added procedural complexity (*n* = 19, 1 had the SRI2 error, 1 had a single missing and flagged allele, and 1 run with a red check). Further, the wet strip failure may have resulted from channel blockage when combined with a lancet. Anchoring methods showed similar first pass rates (76.9% swab, 83.3% lancet, 75% no swab). However, the “with lancet” condition had the lowest total signal averages and may have contributed to a run failure, while “with swab” maintained better consistency between wet and dry treatments (28.4-point difference in average total signal per run). Across sample locations, the middle section of the Bode Collector exhibited the lowest average total signal per run. When the middle was compared to the edge sections (left and right combined), its average total signal per run was significantly lower (t-test comparing average total signal per run, *p* < 0.0001, *n* = 24 edge, *n* = 12 middle). In contrast, the left and right edge sections did not differ from each other (t-test comparing average total signal per run, *p* = 0.21, *n* = 12). Despite these differences, there was no appreciable impact on the first pass success rate when comparing the edges to the middle section.

### Aged samples

**Fig. 6:**
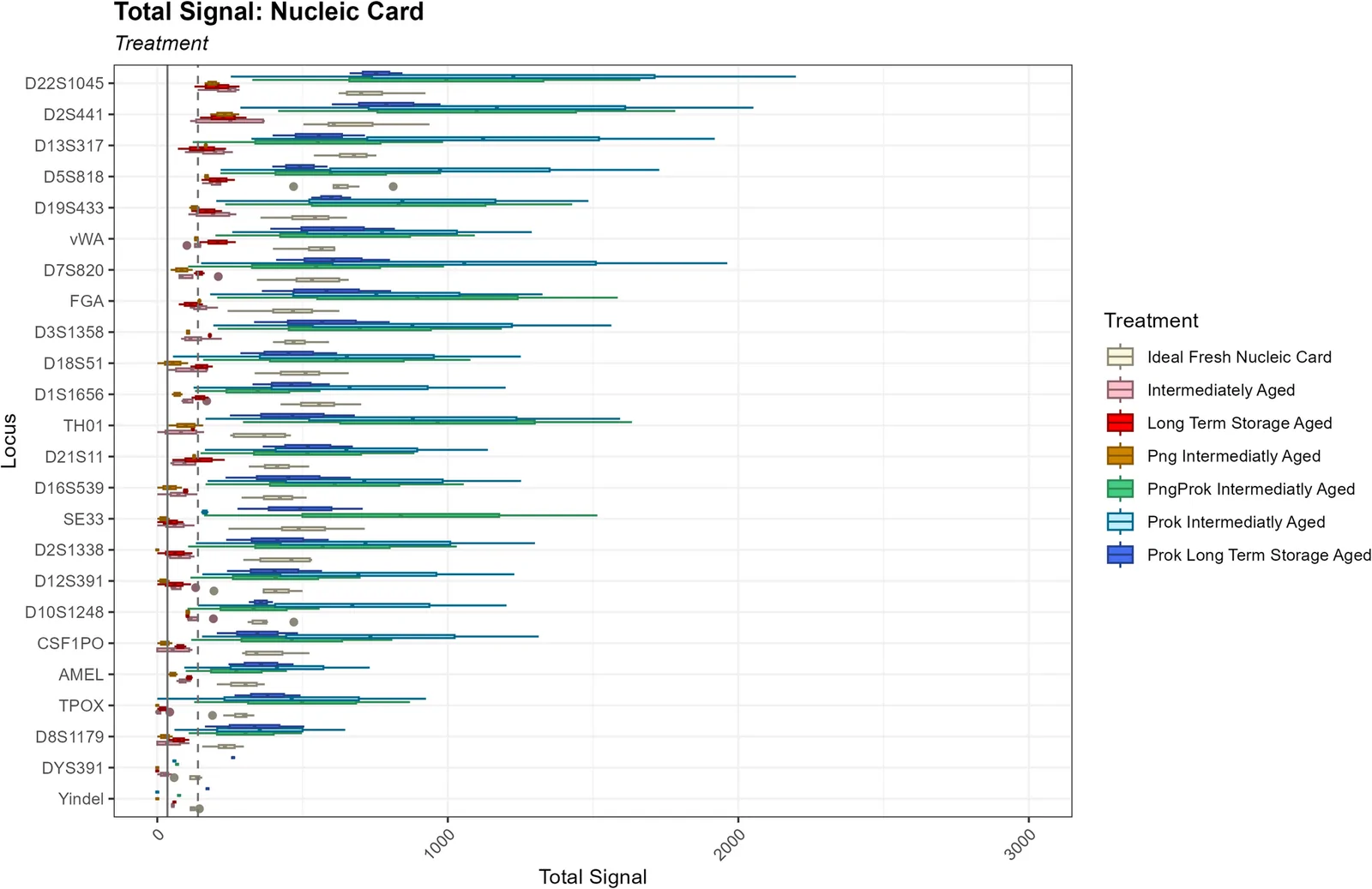
Box plots of total signal (x-axis) by locus (y-axis) for Nucleic-cards™ under various treatment conditions. Treatments include (indicated by color): no additional treatment fresh (pale yellow), no additional treatment intermediately aged (pink), dry long-term storage with no additional treatment (red), Png intermediately aged (dark orange), PngProk intermediately aged (green), Prok intermediately aged (light blue), and Prok long-term storage aged (dark blue). The vertical gray bars show the analytical threshold at 35 RFU (solid gray line) and the stochastic threshold at 140 RFU (dotted gray line)

**Fig. 7:**
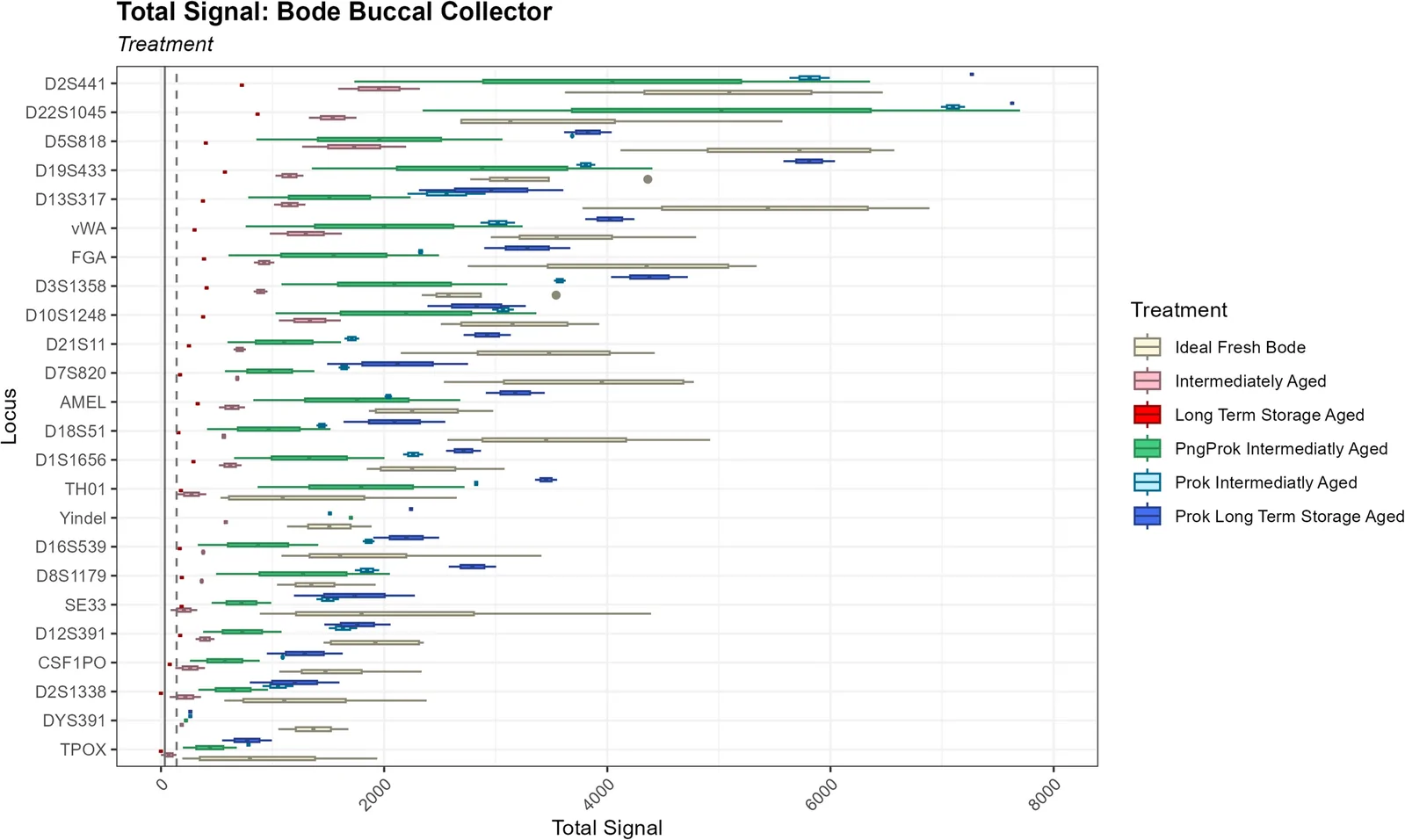
Box plots of total signal (x-axis) by locus (y-axis) for Bode Buccal DNA Collector™ cards across different treatment conditions. Treatments include (indicated by color): no additional treatment fresh (pale yellow), no additional treatment intermediately aged (pink), dry long-term storage with no additional treatment (red), Png intermediately aged (dark orange), PngProk intermediately aged (green), Prok intermediately aged (light blue), and Prok long-term storage aged (dark blue). The vertical gray bars show the analytical threshold at 35 RFU (solid gray line) and the stochastic threshold at 140 RFU (dotted gray line)

**Fig. 8:**
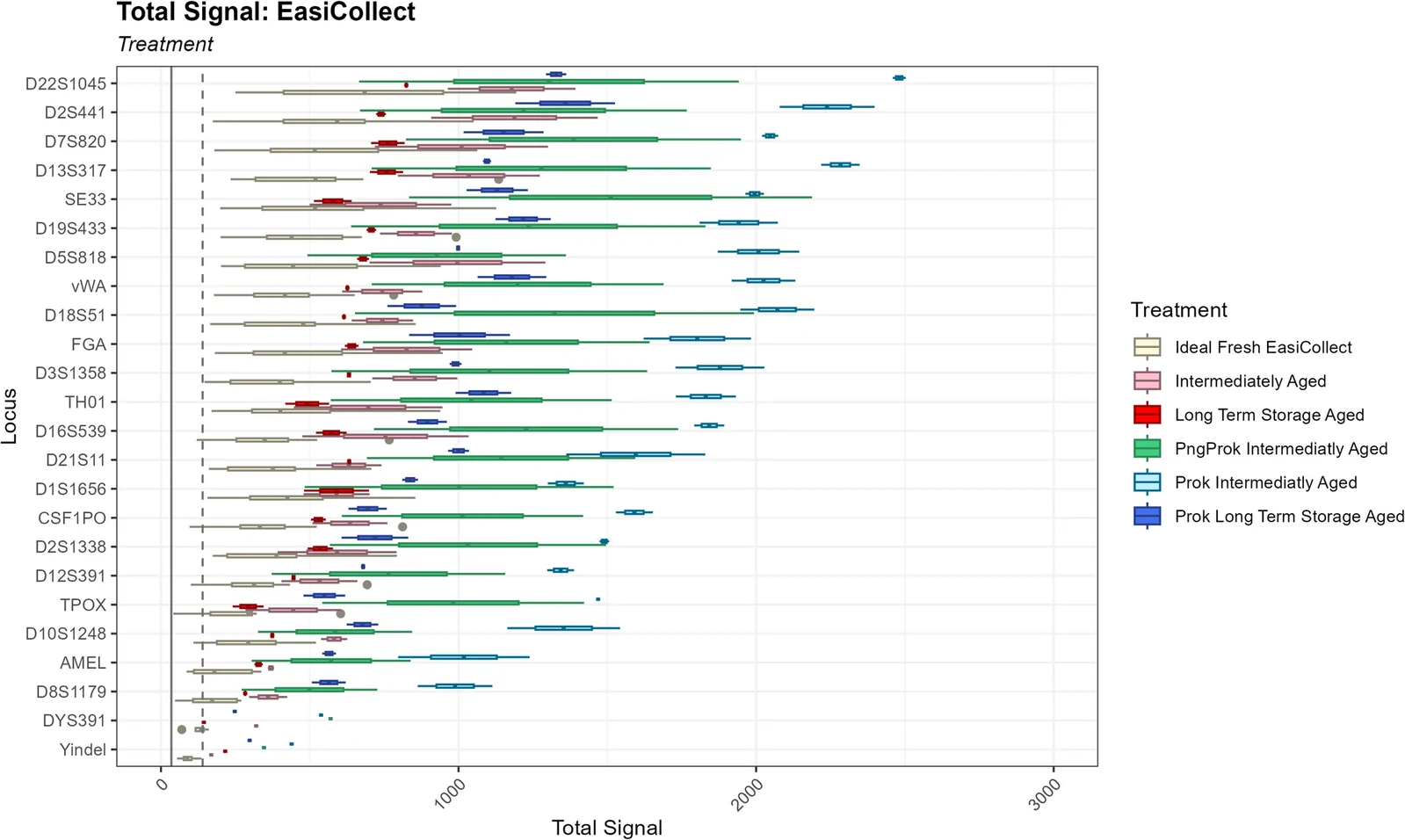
Box plots of total signal (x-axis) by locus (y-axis) for EasiCollect™ devices under different treatment conditions. Treatments include (indicated by color): no additional treatment fresh (pale yellow), no additional treatment intermediately aged (pink), dry long-term storage with no additional treatment (red), Png intermediately aged (dark orange), PngProk intermediately aged (green), Prok intermediately aged (light blue), and Prok long-term storage aged (dark blue). The vertical gray bars show the analytical threshold at 35 RFU (solid gray line) and the stochastic threshold at 140 RFU (dotted gray line)

**Fig. 9:**
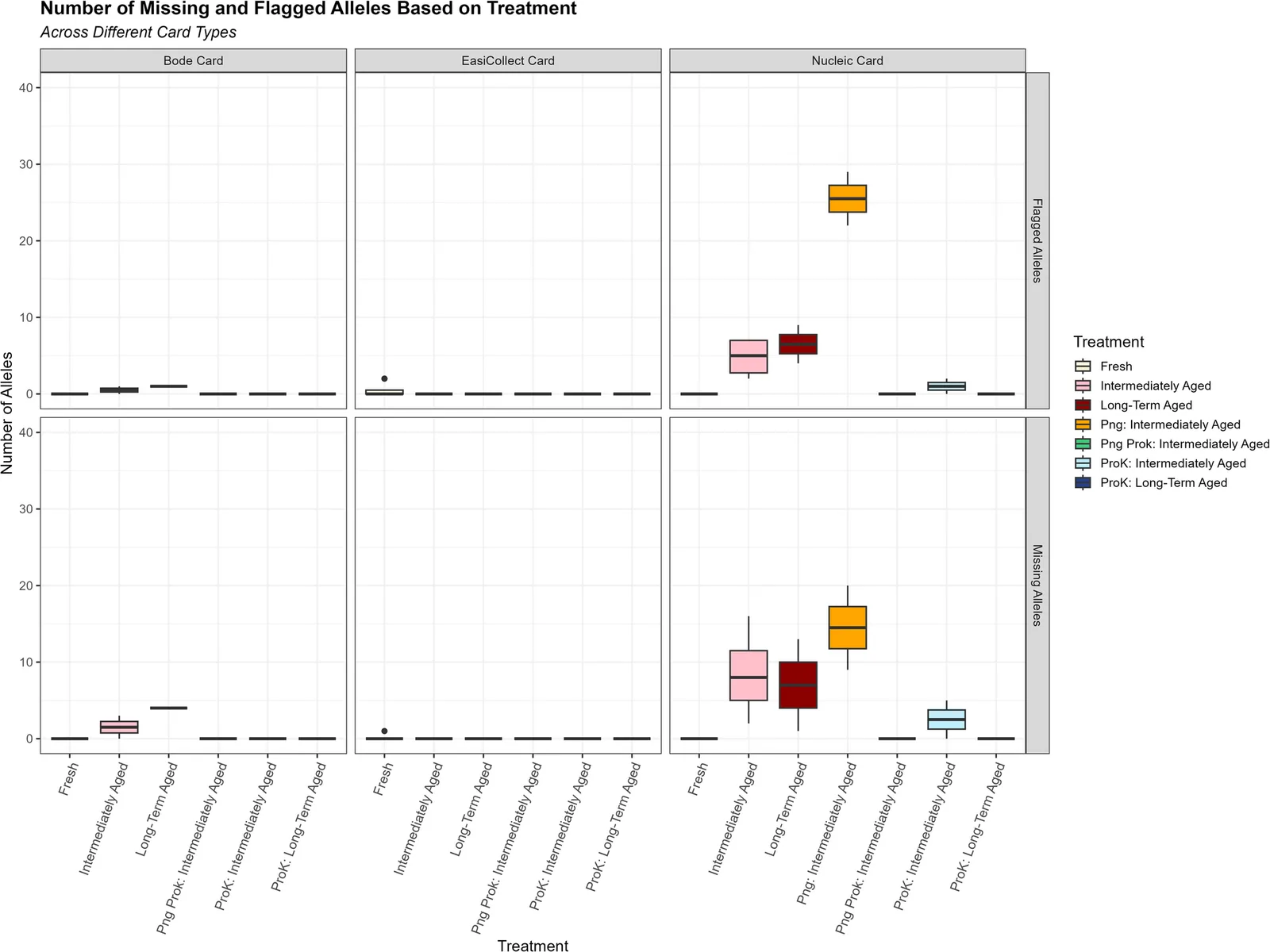
Box plots illustrating the number of flagged and missing alleles (facets; rows) across all DNA collection cards under various treatment conditions. Treatments include (indicated by color): no additional treatment (dry) intermediately aged (pink), dry long-term storage with no additional treatment (red), ideal fresh (pale yellow), Png intermediately aged (dark orange), PngProk intermediately aged (green), Prok intermediately aged (light blue), and Prok long-term storage (dark blue)

This study evaluated DNA cards under three conditions - freshly collected dry (data used from studies described above), intermediately aged (1.5 years on average), and long-term storage (3 years on average)—to identify the optimal treatment for intermediately aged and long-term storage samples. All Bode Buccal DNA Collectors™ in this sub-study were run with no anchor; Nucleic-card™ and EasiCollect™ were both run with a lancet. Due to the time and effort that goes into aging samples for an extended period of time, a minimal number of samples were available for use in this sub-study. Therefore, evaluations were based on a limited number of runs, and the results should be interpreted with this in mind. Supplemental Table 1 shows the average total signal per run and standard deviation for each card type and condition. Nucleic-cards™ (Fig. 6) showed that fresh samples with no additional treatment had an average total signal per run of 416 (SD = 170), above threshold values. Intermediately aged and long-term storage samples with no treatment had lower average total signals of 79 (SD = 75) and 124 (SD = 77.3), respectively, both falling between analytical and stochastic threshold ranges. Similarly, Bode Cards (Fig. 7) showed that fresh samples had an average total signal per run of 2884 (SD = 1565), well above thresholds, while intermediately aged and long-term storage samples showed reduced average total signals of 771 (SD = 555) and 299 (SD = 214), respectively. EasiCollect™ cards (Fig. 8) demonstrated more consistent performance across all sample ages: fresh samples averaged 443 (SD = 251), while intermediately aged and long-term storage samples showed average total signals of 722 (SD = 312) and 557 (SD = 174), respectively. Flagged and missing alleles, shown in Fig. 9, were evaluated across all DNA card types. Untreated Bode Cards showed no flags or dropouts for fresh samples; however, intermediately aged had one sample with 3 missing and 1 flagged allele, and long-term storage samples had one sample with four missing and one flagged allele. EasiCollect™ cards had one fresh sample with 1 missing and 2 flagged alleles, and one sample with 2 flagged alleles; interestingly, the short-term aged samples and the long-term aged samples had zero missing or flagged alleles. Nucleic-cards™ showed no flags or missing alleles for fresh samples, but intermediately aged averaged 13 missing and 7 flagged alleles, and long-term storage samples had an average of 7 missing and 7 flagged alleles. Given that, with the exception of EasiCollect™, the number of flagged and missing alleles increased with sample age, potential treatment options were investigated.

The first treatment tested was punch size. Supplemental Fig. 12 shows the distribution of flagged and missing alleles by punch size for Nucleic-cards™. The previously used 6 mm punch produced a higher average number of flags (7), whereas the 6 mm × 2 punches resulted in fewer flags (1 on average). For missing alleles, the 6 mm × 2 punches averaged 4 alleles, compared to 13 alleles for the 6 mm punch. However, the differences were not considered substantial (i.e., were not consistently producing full, quality flag-free profiles) to justify using twice the amount of sample material. Thus, the use of the single 6 mm punch was retained, especially considering that aged samples are often limited.

After evaluating punch size, the next approach explored applied chemical treatments. The two best-performing treatments from the Nucleic-card™ tests were then applied to the other two card types. This approach conserved aged material and minimized unnecessary consumption. As shown in Fig. 6, intermediately aged Nucleic-cards™ received Proteinase K (Prok) treatment, Prep-n-Go™ buffer (Png) incubated, and Png combined with Prok (PngProk) incubated. Png treatment alone had minimal impact increasing average total signal per run from 79 (SD = 75) to 87 (SD = 71), while PngProk improved the average total signal per run from 79 (SD = 75) to 594 (SD = 530). Prok treatment alone produced the highest average total signal per run from 79 (SD = 75) to 721 (SD = 684). Given Png’s minimal impact on total signal, with it still below stochastic thresholds, it was not applied to the Bode Collectors or EasiCollect™ cards. For Bode Buccal DNA Collector™ cards (Fig. 7), PngProk treatment improved intermediately aged samples’ average total signal per run from 771 (SD = 555) to 1625 (SD = 1541), while Prok treatment restored average total signal per run levels near those of fresh samples with 2500 (SD = 1531). For EasiCollect™ (Fig. 8), Prok treatment of intermediately aged samples generated the highest average total signal per run going from 722 (SD = 312) to 1702 (SD = 480) followed by PngProk at 1042 (SD = 570). Flags and dropouts for treated intermediately aged samples (Fig. 9) showed Bode Cards and EasiCollects™ had no flagged or missing alleles. Nucleic-cards™ treated with Png had more than double the flags compared to the untreated samples, where untreated had an average of 13 missing and flagged alleles and Png had an average of 15 missing and 26 flagged alleles. PngProk-treated samples showed no flags, while Prok-treated samples had a single sample with 5 missing and 2 flagged alleles, which is fewer than untreated intermediately aged counterparts.

Prok treatment (alone) offered the operationally simplest application and greatest increase in total signal. Thus, it was applied to long-term storage samples. For Nucleic-cards™ (Fig. 6), Prok treatment had an average total signal per run of 486 (SD = 212), comparable to no treatment long term aged at 124 (SD = 77). For Bode Cards (Fig. 7), Prok treatment of long-term storage samples resulted in the highest average total signal per run observed going from 299 (SD = 214) at no treatment long term aged to 3156 (SD = 1948) with Prok treatment. For EasiCollect™ cards (Fig. 8), Prok treatment also improved signal output from 577 (SD = 174) to 907 (SD = 289). Flags and dropouts for long-term storage samples (Fig. 9) showed all card types had no flagged or missing alleles post-treatment.

These findings demonstrate that Prok pre-treatment (prior to loading in the sample cartridge) enhances total signal and reduces the number of flagged and/or missing alleles in the generated profile, particularly for intermediately aged and long-term storage samples on Nucleic-cards™ and Bode Cards. This improvement supports its application under a broader range of conditions to improve run quality and DNA profiling reliability. EasiCollects™, on the other hand, performed consistently well (with nearly zero flagged or missing alleles) across all aging conditions regardless of treatment, suggesting it may not require additional treatment.

## Conclusion

This study aimed to evaluate and optimize the use of DNA collection cards on the RapidHIT™ ID System with the RapidHIT™ ID ACE GlobalFiler™ Express Cartridge, providing guidelines and considerations for agencies in diverse operational settings, from laboratories to field environments. Key areas of focus included punch size optimization, preventing cross-contamination, assessing performance under various conditions, and recovering DNA from aged samples.

Sub-study “Cross-Contamination Detection and Punch Size Optimization” evaluated for possible cross-contamination and optimal punch sizes from DNA collection cards for profile generation with the RapidHIT™ ID system. Using the outlined methods, STR profiles exhibited no evidence of cross-contamination between samples or in blanks, suggesting that the described workflow provides a practical approach for minimizing contamination risk. For punch size, several points have to be considered. Especially in a forensic setting, sample availability may be limited, so balancing the need to conserve the sample along with obtaining a quality profile and being operationally practical is important. Smaller punches may conserve the sample card; however, they provide less DNA for the workflow, can be difficult to handle (e.g., prone to static cling), or may not get submerged in the lysis buffer (getting trapped near the top of the sample cartridge or floating on top of the lysis buffer). This sub-study found that the larger punches improved first-pass success rates, with the 6 mm punch size having the fewest flagged and missing alleles under the tested conditions.

Once a punch is collected, transfer to the cartridge and proper placement in the cartridge is needed for a successful run. Using a lancet or another form of anchor can aid in transferring the punch directly into the sample cartridge and ensure it is fully submerged in the lysis buffer. Without an anchor, handling the small punch may be challenging, especially with gloved hands, due to static cling. However, omitting the anchor simplifies the protocol, reduces the need for additional materials, and minimizes the risk of contamination introduced by the anchor itself. Important to consider are the differences between treated and untreated DNA collection cards used in this study, particularly the shape of the punch/strip going into the cartridge, potentially influencing overall performance. Smaller, circular punches were created and used from the treated cards, whereas untreated cards were cut into long rectangular-like strips. This means the different shapes may behave differently once placed in the sample cartridge. Similar to omitting an anchor, not wetting the punch and keeping it dry makes transfer more difficult, as static cling increases the risk of the punch being dislodged or blown away. While wetting the punch improves handling and mitigates static cling, it introduces an extra step and a greater potential for contamination. A final consideration is the ability to rerun the punches in the event a run fails (e.g., a cartridge lot issue, power interruption, or other technical errors) or the sample needs to be rerun with a different workflow or by a different laboratory/agency. In such cases, there is value in determining whether a previously used punch can be rerun and still produce a profile that passes the initial quality check and produces signal above the analytical and stochastic thresholds.

Sub-study “Performance Evaluation of Treated DNA Cards” assessed the performance of treated DNA collection cards (Nucleic-card™ and EasiCollect™) on the RapidHIT™ ID system, examining the effects of wetting, anchoring, and rerun ability. Both cards produced complete, quality flag free profiles during the initial runs. Anchoring with a lancet improved punch placement without a visible impact on total signal. During the initial runs, both cards performed similarly, while EasiCollect’s™ average total signal per run decreased when the rerun punch was dry and maintained total signal when wet. Modified Rapid Analysis allowed for generation of comparable first pass success rates for both the initial and secondary (reruns) runs.

Sub-study “Performance Evaluation of Bode Buccal DNA Collector™ (Untreated DNA Card)” assessed the performance of the untreated DNA collection cards (Bode Buccal DNA Collector™) on the RapidHIT™ ID system, examining treatment, anchoring method, and sample location. Due to shape and manufacturer recommendations, the Bode Collector was cut into three strips, which leads to the possibility that location of the strip relative to the Bode Collector and the strip shape may have downstream implications. Dry treatment had higher or equivalent total signals across all conditions, with greater signal in the left and right sections, possibly due to the placement being on the edge of the collection device where it may pick up more saliva during collection. Anchoring methods showed comparable first pass success rates, but the ‘with lancet’ condition had the lowest average total signals per run where the additional weight from the lancet could have potentially added to blocking the channel or kept the strip tightly folded restricting access to the filter paper surface area. For example, the “wet with lancet” samples had a single run fail, where the error code suggests the wet strip with the lancet anchoring it down could have blocked the channel leading to a run failure. Additionally, the SRI2 error occurred in six runs, resulting in quality flags. When these samples were reprocessed using the current instrument software (V10.4.0.10), five produced complete profiles. This demonstrates that SRI2 errors may require Modified Rapid DNA Analysis; however, they do not necessarily prevent recovery of full profiles.

Sub-study “Aged Samples” evaluated DNA cards under fresh, intermediately aged (1.5 years), and long-term storage (3 years) conditions to optimize treatments for aged samples. In real-world workflows, samples may remain in storage due to backlogs, reagent or cartridge shortages, or the need for reruns. Further, samples may be stored under varying conditions, including (for example) room temperature, refrigeration, or freezing. As samples age, DNA quantity and quality can decline, which can impact STR profile generation. In this study, samples were stored at room temperature to establish a baseline and focus on workflow requiring minimal equipment and storage needs; however, it is possible that refrigeration or frozen storage may mitigate some of the signal reduction observed. Retaining samples for extended periods (1.5–3 years) can be challenging, resulting in a limited number of aged samples avaliable for this sub-study. For the data produced herein, with the exception of the EasiCollect™ card, which remained consistent, aging was associated with a decrease in average total signal per run and an increase in the number of missing or flagged alleles. Adding an additional ProK treatment step increased average total signal per run and reduced flagged or missing alleles in Nucleic-card™ and Bode samples, particularly in intermediately aged and long-term storage conditions. While the additional pre-treatment improved the performance of aged samples, laboratories considering this option must evaluate the feasibility of this step in their own context (i.e., Do I have the equipment, storage capacity, and reagents at my Rapid DNA instrument’s location to facilitate this extra step? ).

This study explored several workflow factors that may influence profile quality when using DNA collection cards with the RapidHIT™ ID system, including punch size, handling methods, and treatment options for aged samples. A total of 205 runs were conducted across various conditions, providing observations that may help inform laboratory workflows. These findings may assist those interested in RapidHIT™ ID system in developing internal guidelines for the use of DNA collection cards and provides a basis for future studies.

## Electronic Supplementary Material

Below is the link to the electronic supplementary material.

Supplementary File 1
