# Supplementary material for: Optimization of DNA collection card workflows on the RapidHIT™ ID instrument
Source: Int J Legal Med. 2026 Jun 26;140(6):3649–65. doi: 10.1007/s00414-026-03885-z (PMC13658342; doi:10.1007/s00414-026-03885-z)
Supplement: Supplementary file 1 [file 414_2026_3885_MOESM1_ESM.docx]

**Supplemental Information DNA Collection Cards Paper**

**Supplemental Figure 1.**

Pictures of Nucleic-card™ punches at three different sizes weighed down by a lancet and run on the RapidHIT™ ID instrument. The three punch sizes included a 3 mm punch (A), two 3 mm punches (B), and a 6 mm punch (C).

**Supplemental Figure 2.**

Pictures illustrating a 6 mm punch loaded into an Ace GlobalFiler™ cartridge without (A) and with (B) a lancet to assist with positioning and weighing the punch down.

**Supplemental Figure 3.**

Pictures illustrating a Bode Buccal DNA Collector™ being cut into 5 mm strips (A) and secured in the Ace GlobalFiler™ cartridge with a swab (B).

**Supplemental Figure 4.**

STR profiles generated by the RapidHIT™ ID system of two blank runs and a DNA positive run. In the AMEL locus, run blank (A) has an uncalled artifact, run blank (B) has a peak flag labeled as "OB,” and DNA positive sample (C) has an uncalled artifact. A black arrow and circle are used to highlight the area of interest.

**Supplemental Figure 5.**

STR profile generated by the RapidHIT™ ID system depicting a mixture between the female and male subject (A). STR profile of the female (B) and male (C) subject with no contamination observed.

**Supplemental Figure 6.**

Box plot illustrating the number of flagged and missing alleles (facets; columns) across treated DNA collection cards (facets; rows) separated by run order (x-axis). Runs included (indicated by color): initial run, non-modified rerun, and modified rerun.

**Supplemental Figure 7.**

Bar graphs illustrating the first pass success rates (color) for the applied conditions (x-axis; dry no lancet, dry with lancet, and wet with lancet) for EasiCollect™ Devices and Nucleic-cards™ (facets). Green bars indicate runs with no quality flags, while yellow bars indicate runs with at least one flag.

**Supplemental Figure 8.**

Box plot illustrating the number of flagged and missing alleles across treated DNA collection cards (facets; rows) separated by anchoring method (x-axis). Anchoring methods included (indicated by color): no swab, wet treatment with a lancet, and dry treatment with a lancet.

**Supplemental Figure 9.**

Box plots of total signal for EasiCollect™ Devices by treatment and anchoring (facets; rows). Loci are indicated on the y-axis and total signal on the x-axis. The runs that received the wetting protocol are dark blue, and the dry samples are light blue. The vertical gray bars show the analytical threshold at 35 RFU (solid gray line) and stochastic thresholds at 140 RFU (dotted gray line). The vertical solid black bar represents the average total signal.

**Supplemental Figure 10.**

Box plots of total signal for Nucleic-card™ by treatment and anchoring (facets; rows). Loci are indicated on the y-axis and total signal on the x-axis. The runs that received the wetting protocol are dark blue, and the dry samples are light blue. The vertical gray bars show the analytical threshold at 35 RFU (solid gray line) and stochastic thresholds at 140 RFU (dotted gray line). The vertical solid black bar represents the average total signal.

**Supplemental Figure 11.**

Picture illustrating a Bode Buccal DNA Collector™ with a 5 mm strip secured in the sample cartridge with a lancet, where lysate is visible in the Ace GlobalFiler™ cartridge.

**Supplemental Table 1.**

Direct comparison of average total signal between treatment, age, and DNA collection card type. Values are presented as average total signal and standard deviation (SD) for each condition.

|  | Nucleic-card™ | Bode Buccal DNA Collector™ | EasiCollect™ Devices |
| --- | --- | --- | --- |
| Ideal Fresh | Average = 416  SD = 170 | Average = 2884  SD = 1565 | Average = 433  SD = 251 |
| Intermediately Aged | Average = 79  SD = 75 | Average = 771  SD = 555 | Average = 722  SD = 312 |
| Long Term Storage Aged | Average = 124  SD = 77 | Average = 299  SD = 214 | Average = 557  SD = 174 |
| Png Intermediately Aged | Average = 87  SD = 71 |  |  |
| PngProk Intermediately Aged | Average = 594  SD = 530 | Average = 1625  SD = 1541 | Average = 1042  SD = 570 |
| Prok Intermediately Aged | Average = 721  SD = 684 | Average = 2500  SD = 1531 | Average = 1702  SD = 570 |
| Prok Long Term Storage Aged | Average = 486  SD = 212 | Average = 3156  SD = 1948 | Average = 907  SD = 289 |

**Supplemental Figure 12.**

Box plots illustrating the number of flagged and missing alleles (facets) on aged Nucleic-cards™ by punch size (x-axis). The 6 mm punch is tan in color, and the 6 mm x 2 is brown in color.
